# Supplementary figures and images for: A Role for the VPS Retromer in Brucella Intracellular Replication Revealed by Genomewide siRNA Screening
Source: mSphere. 2019 Jun 26;4(3):e00380-19. doi: 10.1128/mSphere.00380-19 (PMC6595151; doi:10.1128/mSphere.00380-19)

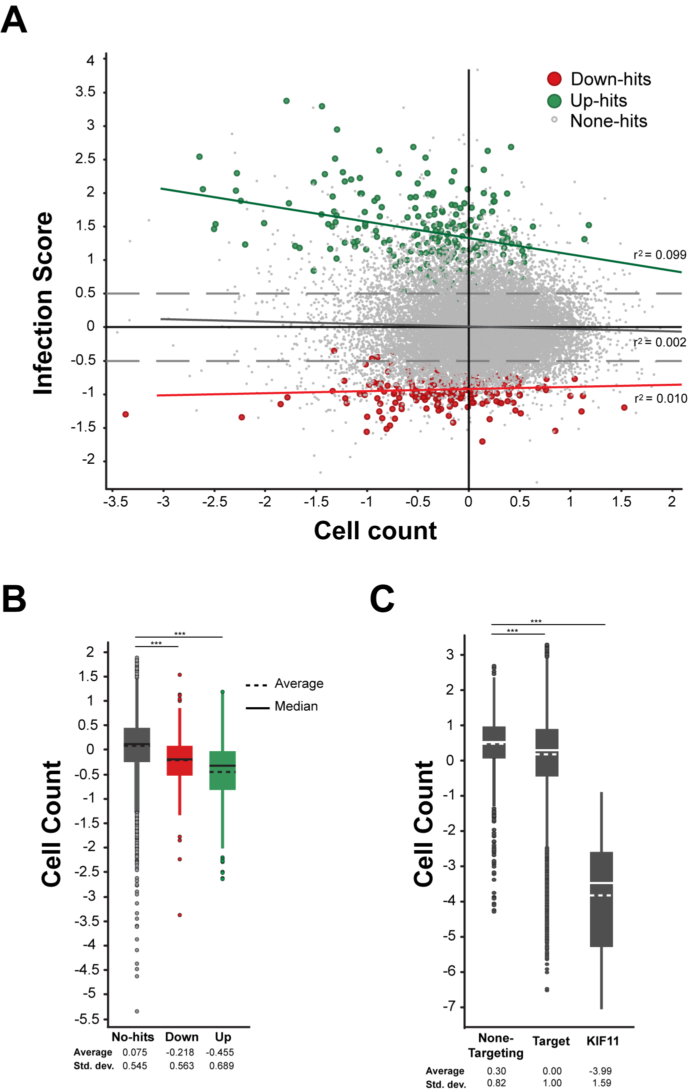

Supplement: FIG S1 [file mSphere.00380-19-sf001.tif]
