# Supplementary material for: A Role for the VPS Retromer in Brucella Intracellular Replication Revealed by Genomewide siRNA Screening
Source: mSphere. 2019 Jun 26;4(3):e00380-19. doi: 10.1128/mSphere.00380-19 (PMC6595151; doi:10.1128/mSphere.00380-19)
Supplement: TABLE S4 [file mSphere.00380-19-st004.pdf]

**Table S4**

| | VPS35 | | | VPS29 | | | VPS26 | | |
|---|---|---|---|---|---|---|---|---|---|
| **siRNA** | AVG | | SD | AVG | | SD | AVG | | SD |
| **siVPS35** | **21.5** | **±** | **2.4** | 25.8 | ± | 11.5 | 25.1 | ± | 6.2 |
| **siVPS29** | 105.8 | ± | 10.4 | **6.6** | **±** | **5.3** | 90.2 | ± | 36.8 |
| **siVPS26A** | 65.5 | ± | 13.9 | 21.1 | ± | 21.5 | **10.6** | **±** | **2.9** |
| **siVPS26A/B** | 49.5 | ± | 12.9 | 23.7 | ± | 31.3 | **5.5** | **±** | **3.3** |
